# Case Report: A Novel *CACNA1S* Mutation Associated With Hypokalemic Periodic Paralysis in a Chinese Family

**DOI:** 10.3389/fgene.2021.743184

**Published:** 2021-10-29

**Authors:** Jie-Yuan Jin, Bing-Bing Guo, Yi Dong, Yue Sheng, Liang-Liang Fan, Li-Bing Zhang

**Affiliations:** ^1^ School of Life Sciences, Central South University, Changsha, China; ^2^ Hunan Key Laboratory of Animal Models for Human Diseases, School of Life Sciences, Central South University, Changsha, China; ^3^ CAS Key Laboratory of Infection and Immunity, Institute of Biophysics, Chinese Academy of Sciences, Beijing, China; ^4^ Hunan Key Laboratory of Medical Genetics, School of Life Sciences, Central South University, Changsha, China; ^5^ Department of Pediatrics, Affiliated Hospital of Yangzhou University, Yangzhou, China

**Keywords:** CACNA1S, hypokalaemic periodic paralysis, frameshift mutation, calcium channels, targeted sequencing

## Abstract

Hypokalemic periodic paralysis (HypoPP) is a rare autosomal dominant disorder characterized by episodic flaccid paralysis with concomitant hypokalemia. More than half of patients were associated with mutations in *CACNA1S* that encodes the alpha-1-subunit of the skeletal muscle L-type voltage-dependent calcium channel. Mutations in *CACNA1S* may alter the structure of CACNA1S and affect the functions of calcium channels, which damages Ca^2+^-mediated excitation-contraction coupling. In this research, we identified and described a Chinese HypoPP patient with a novel frameshift mutation in *CACNA1S* [NM_000069.2: c.1364delA (p.Asn455fs)] by targeted sequencing. This study would expand the spectrum of *CACNA1S* mutations, further our understanding of HypoPP, and provided a new perspective for selecting effective treatments.

## Introduction

Hypokalemic periodic paralysis (HypoPP) is a rare neuromuscular disorder with an estimated prevalence of 1/100,000 (Hirano et al., 2011). HypoPP, hyperkalemic paralysis (HyperPP) and Andersen-Tawil syndrome (ATS) constitute the dominant types of primary periodic paralysis (PPs) (Statland et al., 2018). Its most prominent characteristic is partial or systemic episodic severe muscle weakness occurring in association with hypokalemia (<3.5 mEq/L). The occurrence of the first attack is usually within the first or second decade, and every attack can last from hours to days (Houinato et al., 2007). Generally, it is triggered by the before rest after vigorous exercise, carbohydrate-rich diets, and exposure to heat or cold. The degree of paralysis is variable, patients usually present with paralysis in the proximal muscle groups of the limbs. Under worse conditions, some may die of respiratory paralysis or arrhythmia (Alhasan et al., 2019).

HypoPP follows an autosomal dominant pattern with incomplete penetrance, especially in women (Hirano et al., 2011). Molecular genetic analyses have revealed that it is caused by mutations in *CACNA1S* and *SCN4A*. In other PPs types, ATS is associated with KCNJ2 defects, and HyperPP is due to gain-of-function *SCN4A* mutations (Krych et al., 2017; Tan et al., 2020). Other rare genes (such as *KCNJ5* and *ATP2A1*) have been reported to be linked with PPs (Shull et al., 2003; Arzel-Hezode et al., 2009). Up to 80% of HypoPP cases are attributed to *CACNA1S* mutations (Ke et al., 2013). *CACNA1S* is located on chromosomes 1q31–q32 encoding the *α*-subunit of skeletal muscle voltage-gated calcium channel (Cav1.1) (Ke et al., 2009). CACNA1S is primarily distributed in the membrane of the transverse tubular system and is involved in Ca^2+^-mediated excitation-contraction coupling (Kil and Kim, 2010). *CACNA1S* mutations may break the balance of electric potential. In fact, gating pore currents alteration is a widely accepted mechanism to explain the occurrence of HypoPP in most Cav1.1 mutation cases (Wu et al., 2012).

In this study, we reported a Chinese family with HypoPP. A novel frameshift mutation c.1364delA (p.Asn455fs) in *CACNA1S* is responsible for the disease in this family. This study would expand the spectrum of *CACNA1S* mutations, further our understanding of HypoPP, and provide a new perspective for selecting effective treatments.

## Materials and Methods

### Patients and Subjects

The study participants were enrolled in Affiliated Hospital of Yangzhou University. This study was approved by the Review Board of Affiliated Hospital of Yangzhou University. Written informed consent was obtained from the proband and his guardian. Detailed records of family medical history, physical examinations, and presentations and features including blood biochemistry, blood gas analysis, thyroid function, and routine urine examination were obtained from the proband to exclude other causes of hypokalemia.

### Deoxy-ribo Nucleic Acid Extraction

Genomic DNA was extracted from peripheral blood samples of the proband and his family using the DNeasy Blood and Tissue Kit (Qiagen, Valencia, CA, United States).

### Targeted Sequencing

Targeted sequencing was performed in the proband. A panel of 143 common nuclear genes (Supplementary Table S1), including known endocrine system genetic diseases-related genes, was captured by the SureSelectXT2 Target Enrichment System (Agilent, Santa Clara, CA, United States) according to reported methods. After enrichment, libraries were sequenced using a HiSeq X-10 system (Illumina, San Diego, CA, United States). All variants were analyzed using the SureCall software (Agilent, Santa Clara, CA, United States). Variants with a mean coverage of ≥100 were taken into consideration. After filtering the common variants (frequency ≥0.01) based on the 1,000 Genomes Project database (1,000G; https://www.genome.gov/27528684/1000-genomes-project/) and the Genome Aggregation Database (GnomAD; http://gnomad.broadinstitule.org), unique single-nucleotide polymorphisms (SNPs) were detected in the subject. These variants were predicted using bioinformatics programs including MutationTaster (http://www.mutationtaster.org/), Polyphen-2 (http://genetics.bwh.harvard.edu/pph2/), and SIFT (http://provean.jcvi.org/index.php). Gene function, inheritance pattern, clinical phenotype, and pathogenicity were annotated according to the Online Mendelian Inheritance in Man (OMIM; https://www.omim.org) and American College of Medical Genetics (ACMG) classification (Richards et al., 2015).

### Co-Segregation Analysis

Primer pairs (CACNA1S f: 5′-CTA​CGC​ATG​CCT​GGA​GTT​T-3’; r: 3′-TGG​TGC​CAT​TGG​CTG​ATT-5′) were designed by Integrated DNA Technologies (https://sg.idtdna.com/pages). The target fragment was amplified by polymerase chain reaction (PCR) and analyzed using the ABI 3100 Genetic Analyzer (ABI, Foster City, CA, United States).

## Results

### Case Description

A 12-year-old boy (II-1) was admitted to our hospital complaining of weakness in both lower limbs after falling from a height of two steps after school on September 2, 2020. During this episode, the symptoms exacerbated when he was climbing stairs. The proband could walk independently on flat ground at a slow pace after getting up in the morning but was unable to walk without support later. The electrolyte test indicated that the serum potassium level was 2.43 mmol/L (normal range, 3.5–5.5 mmol/L). During the attack of weakness, he exhibited the absent of the tendon reflexes, without interictal opthalmoplegia or facial weakness. At this point, based on Manual Muscle Testing (MMT), his baseline strength of right and left lower extremity were grade 1/0, while upper limbs were grade 4/4-.

Other vital signs and parameters, including thyroid function, calcium and magnesium levels, urinary electrolytes, aldosterone, and plasma renin activity, were all within normal ranges. However, levels of insulin (INS; 179.70 pmol/L), creatine kinase (CK; 336.0 U/L) and lactate (LAC; 2.29 mmol/L) were found to were mildly elevated. Pituitary magnetic resonance imaging scan (MR) and adrenal computed tomography scan (CT) showed no abnormalities, no loss of potassium in the digestive tract and skin, and no manifestations of renal tubular acidosis, aldosterone/renin, and thyroid function disorders.

Based on clinical presentations and features, physical inspection, and blood potassium level, the patient was diagnosed with possible HypoPP and was administered an oral and intravenous infusion of potassium supplementation in combination with potassium-preserving diuretic (spironolactone). The treatment alleviated his symptoms (baseline strength of lower limbs becoming grade 5/4+) and restored his potassium level. The phenotypes, treatments, biochemical data, and real-time data on serum potassium levels were outlined in Tables 1, 2. After 10 days of treatment, the patient was discharged with normal baseline strength of limbs. However, the individual reappeared with similar symptoms a month later.

**TABLE 1:** Laboratory data on the first admission.

Blood chemistry	Value	Endocrinology	Value
Na (mmol/L)	143.5(135–148)	Cortisol (µg/dL)	18.39(4.0–18.3)
K (mmol/L)	2.43(3.5–5.0)	Ald (pg/ml)	120.08(30–159)
Cl (mmol/L)	102.7(98–108)	ACTH (pg/ml)	45.37(9–52)
Ca (mmol/L)	2.24(4.1–4.6)	Angiotensin II (pg/ml)	122.38
Pi (mmol/L)	1.27(2.9–4.8)	Renin (pg/ml)	5.14
Mg (mmol/L)	0.80(0.74–1.0)	—	—
HCO_3_^−^ (mmol/L)	24.3(22–27)	—	—
CK (U/L)	336.0(48–208)	—	—
LAC (mmol/L)	2.29(0.5–1.7)	—	—
ALP (U/L)	172(<500)	—	—
LDH (U/L)	280.0(264–437)	—	—
INS (pmol/L)	179.70(29–172)	—	—
C-P (pmol/L)	1,312.69(298–2,350)	—	—
CRP (mg/L)	2.51(<5)	—	—
TG (mmol/L)	3.15(0.02–1.21)	—	—

Parenthesis shows reference range. Red highlights the values out of exception. CK: creatin kinase, LAC: Lactic acid, ALP: Alkaline phosphatase, LDH: lactic dehydrogenase, INS: insulin, C-P: C – peptide, CRP: C-reactive protein, TG: triglyceride, Ald: aldosterone, ACTH: Adrenocorticotropic hormone.

**TABLE 2:** Change in serum potassium values over time.

Date	Serum potassium (mmol/L)
3 September 2020, 12:46	2.43
3 September 2020, 13:33:56	2.24
3 September 2020, 16:28:03	2.32
3 September 2020, 21:37:07	2.26
4 September 2020, 01:50:51	2.65
4 September 2020, 07:12:53	3.47
5 September 2020, 02:23:14	3.91
5 September 2020, 08:48:53	4.05
7 September 2020, 10:36:53	4.65
11 September 2020, 07:05:54	4.09

**FIGURE 1:**
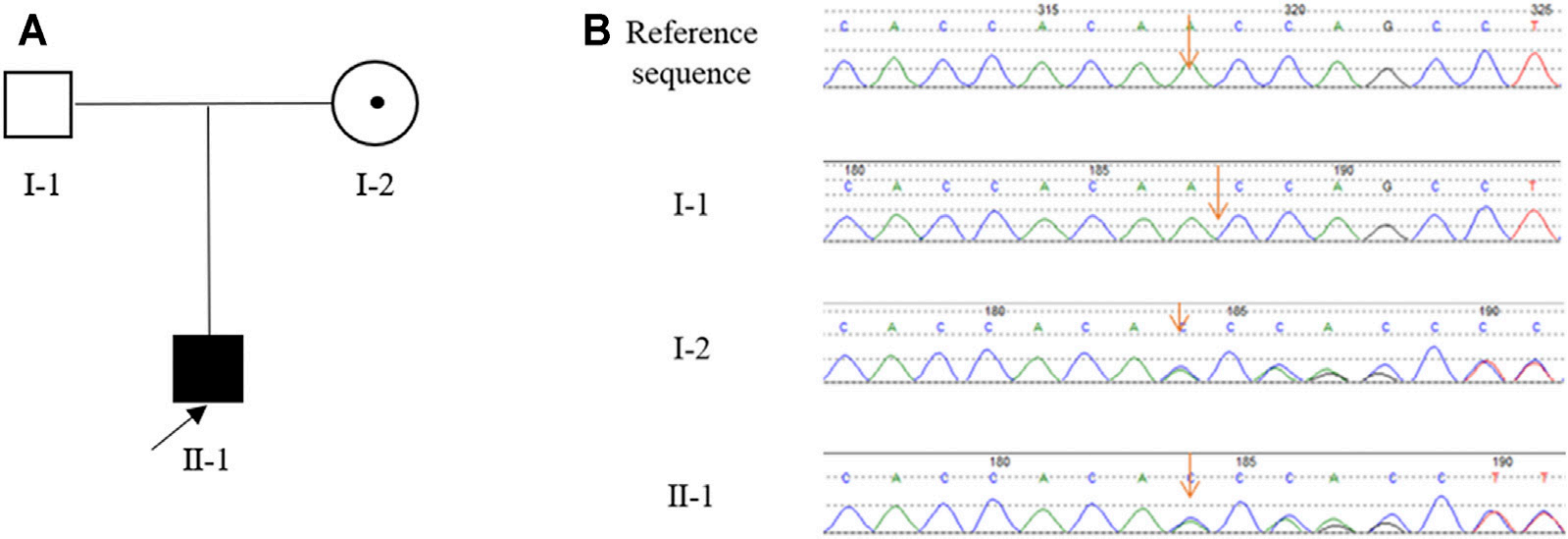
The pedigree of the Chinese family with HypoPP and the result of Sanger sequencing. **(A)**, The pedigree of the Chinese family. The black box represents the HypoPP individual; the black point represents the myasthenic phenotype. **(B)**, Analysis of the DNA sequence. Electropherograms show the sequence encompassing the heterozygous frameshift mutation c.1364delA (p.Asn455fs) in *CACNA1S* in the proband (II-1) and his mother (I-2).

Tracing back the family history of the proband, his mother was once investigated for an episode described as generalised fiatuge. Her acetylcholine receptor antibody test was negative and her serum potassium was normal. Electrophysiology was not performed. The proband’s father was unaffected by paralytic clinical signs in their lifetime. Besides, there was no history of other genetic or infectious diseases in his family (Figure 1A).

### Identification of a Novel *CACNA1S* Mutation Associated With HypoPP

As we reasoned that the proband had been affected by HypoPP based on clinical features and laboratory data, genetic testing of genes related to diseases of the endocrine system genetic diseases was subsequently performed. Using high-throughput sequencing technology, 139 common nuclear genes were examined and the point mutations and CNVs of nuclear genes were filtered. We detected a variant in *CACNA1S* [NM_000069.2: c.1364delA (p.Asn455fs)], which was absent from 1,000G and GnomAD, and predicted to be damaging by MutationTaster, Polyphen-2, and SIFT. Therefore, we suspected that the variant was responsible for HypoPP in this proband.

Sanger sequencing revealed that the proband (II-1) harbored the *CACNA1S* variant which was inherited from his mother (I-2) (Figure 1B). This variant is predicted to cause the frameshift from amino acid 455 of CACNA1S, leading to premature termination of translation and impairment of its function. According to the ACMG guidelines with the following evidence: PVS1, PM2, PM4, PP1, PP4, and PP5, the *CACNA1S* variant was classified as “likely pathogenic”. In line with this, we considered that *CACNA1S* is to be the genetic etiology in the family.

## Discussion

The individual (II-1) in this study fulfilled to the supportive diagnostic criteria for HypoPP (listed in the Supplementary Table S2) (Neame et al., 2017; Statland et al., 2018). The patient had normal intrauterine development and birth, and his mother said with certainty that he was able to participate in sport normally like other peers in the early development. However, the first attack occurred in the second decade of life and was associated with a low serum potassium level (<3.5 mEq/L). The patient showed considerable improvement after potassium intake. Also, there were no other causes of hypokalemia, such as renal, adrenal, or thyroid dysfunction. Clinical myotonia, which is more in Hyperkalemic periodic paralysis but not usually present in HypoPP, was also not observed in the boy (Jurkat-Rott et al., 2000). We also found that the serum CK level is slightly increased. In recent reports, some patients with HypoPP also revealed an uncommon condition of sustained elevation of CK. The elevation of CK was considered an asymptomatic sign of myopathy, which might indirectly explain the phenomenon of muscle weakness (Kurokawa et al., 2020).

The diagnosis of HypoPP can be confirmed by genetic testing after clinical investigations. In this study, we identified a novel heterozygous mutation, c.1364delA (p.Asn455fs), in *CACNA1S* in the proband by targeted sequencing. *CACNA1S* is one of the disease-causing genes involved in HypoPP. HypoPP is an autosomal dominant disorder that shows incomplete penetrance in women (Yang et al., 2015). According to the genetic testing results, the mutation was identified in the mother (I-2), who had an attack of muscle weakness without evident hypokalemia, this may be attributed to the reduced penetrance in female carriers. Several HypoPP mutations, including the *CACNA1S* Arg528His, Arg900Ser, and *SCN4A* Arg672Cys, have been reported to be incomplete-penetrance in women (Kawamura et al., 2004; Kim et al., 2004; Ke et al., 2009; Ke et al., 2015). Skeletal muscle is an amazingly plastic tissue, capable of compensating lesser functional aberrations. Sex hormones act as ion channel regulators with genomic and non-genomic patterns. The differential effects of sex hormones on ion channels may be a factor underlying the variation in penetrance of HypoPP but no study verifies it (Ke et al., 2013). For further determining that CACNA1S was the genetic etiology of the family, we excluded other periodic paralysis causing genes and myopathy-related genes.

Since a high-throughput sequencing approach greatly improves the genetic diagnosis of diseases at the individual or population level, several *CACNA1S* missense mutations have been validated in HypoPP families. Many mutations are substitutions of the positively-charged arginine (R) in S4 within a voltage sensor domain by a non-charged residue. However, no frameshift mutation has been reported to be associated with HypoPP. Only a handful of nonsense, frameshift, or splice-site mutations was found in congenital myopathy families (Hunter et al., 2015; Schartner et al., 2017; Kubota et al., 2020). We may detect the first alone frameshift mutation of *CACNA1S* in HypoPP cases without congenital myopathy phenotype, which may contribute to further understanding the genetic etiology of this disease.

The Cav1.1 complex consists of the pore-forming subunit *α*1 and auxiliary subunits *α*2*δ*, *β*, and *γ*. The auxiliary subunit *β* binds to the cytoplasmic loop between repeats I and II of the *α*1 subunit to modulate membrane trafficking and kinetic properties of the Cav1.1 complex (Flucher, 2020). Other membrane-targeting signals have also been defined in the C-terminus region of the *α*1 subunit, but they are unable to drive the III-IV domain to the cell surface adequately in the absence of the I-II fragment (Flucher et al., 2000; Proenza et al., 2000). The pore-forming subunit *α*1 interacts with the Ca^2+^ release channel (ryanodine receptor; RyR1) by the loop between domains II and III, and the S4 segment of each transmembrane domain acts as a voltage sensing domain (VSD) for the calcium channel. Upon depolarization, S4 segment translocates through a “gating pore” pathway formed by S1-3 segments Li et al. (2012). Activation of the dihydropyridine receptor (DHPR) induces the opening of RyR1 and the release of Ca^2+^ from the sarcoplasmic reticulum stores, subsequently triggering muscle contraction (Block et al., 1988). Changes in the structure may alter the calcium current amplitude or density. HypoPP mutations are gathered in the S4 VSD and have been found in VSDs of I to IV, and CACNA1S mutation p.Asn455fs damaged these domains.

The mechanism by which truncated Ca^2+^ channel proteins may affect muscle function remains unclear. Ahern et al. (2001a) established the following facts: 1) A truncated channel derived from a frameshift mutation may not be functional because it would not traffic to the membrane; or 2) In the condition that another starting codon is present near the frameshift, it may be able to traffic to the membrane. For instance, a frame-shift mutant (fs-alpha1S) expressed the N-terminal half of alpha1S (M1 to L670) and the C-terminal half starting at M701 which was generated by an unexpected restart of translation of the fs-alpha1S message at M701 (Ahern et al., 2001a; Ahern et al., 2001b). Their study further mentioned that the frameshift mutant with deleted residues (Thr671-Leu690) in the cytosolic loop between repeats II and III of *α*1S expressed the N-terminal half (*α*1S 1–670) and the C-terminal half (*α*1S 701–1873) separately. Co-expression of the two fragments resulted in complete recovery of intramembrane charge movement across the DHPR and voltage-induced Ca^2+^ transients in dysgenic myotubes (Ahern et al., 2001b). In our case, the 455Leu residue in exon 10 was located within the domain II S1-2 linker of CACNA1S. The c.1364delA mutation is predicted to cause a frameshift from amino acid 455 of CACNA1S, leading to premature termination of translation (Schartner et al., 2017). Whether re-initiation of translation after a stop signal would apply here is uncertain. Furthermore, premature stop codons may trigger a non-sense-mediated mRNA decay leading to a reduction of CACNA1S. However, we have not yet clarified its biological function and further studies need to be performed.

## Conclusion

In summary, we identified a novel heterozygous mutation, c.1364delA (p.Asn455fs), in *CACNA1S* in a Chinese family with HypoPP. The discovery of this novel mutation would not only enrich the genetic map of HypoPP but also assist in studying the pathogenesis and genetic mechanisms of the disease, providing a new perspective for prevention and treatment.

## Data Availability

The data presented in the study are deposited in the (BioSample) repository, accession number: PRJNA771220, https://www.ncbi.nlm.nih.gov/sra/PRJNA771220.

## References

[B1] Ahern C. A. Arikkath J. Vallejo P. Gurnett C. A. Powers P. A. Campbell K. P. (2001a). Intramembrane Charge Movements and Excitation- Contraction Coupling Expressed by Two-Domain Fragments of the Ca2+ Channel. Proc. Natl. Acad. Sci. 98 (12), 6935–6940. 10.1073/pnas.111001898 11371610 PMC34456

[B2] Ahern C. A. Vallejo P. Mortenson L. Coronado R. (2001b). Functional Analysis of a Frame-Shift Mutant of the Dihydropyridine Receptor Pore Subunit (alpha1S) Expressing Two Complementary Protein Fragments. BMC Physiol. 1, 15. 10.1186/1472-6793-1-15 11806762 PMC64647

[B3] Alhasan K. A. Abdallah M. S. Kari J. A. Bashiri F. A. (2019). Hypokalemic Periodic Paralysis Due to CACNA1S Gene Mutation. Nsj 24 (3), 225–230. 10.17712/nsj.2018.3.20180005 PMC8015512 31380823

[B4] Arzel-Hézode M. McGoey S. Sternberg D. Vicart S. Eymard B. Fontaine B. (2009). Glucocorticoids May Trigger Attacks in Several Types of Periodic Paralysis. Neuromuscul. Disord. 19 (3), 217–219. 10.1016/j.nmd.2008.12.008 19201608

[B5] Block B. A. Imagawa T. Campbell K. P. Franzini-Armstrong C. (1988). Structural Evidence for Direct Interaction between the Molecular Components of the Transverse Tubule/sarcoplasmic Reticulum junction in Skeletal Muscle. J. Cel Biol 107 (6 Pt 2), 2587–2600. 10.1083/jcb.107.6.2587 PMC2115675 2849609

[B6] Flucher B. E. Kasielke N. Grabner M. (2000). The Triad Targeting Signal of the Skeletal Muscle Calcium Channel Is Localized in the Cooh Terminus of the α1S Subunit. J. Cel Biol 151 (2), 467–478. 10.1083/jcb.151.2.467 PMC2192640 11038191

[B7] Flucher B. E. (2020). Skeletal Muscle CaV1.1 Channelopathies. Pflugers Arch. - Eur. J. Physiol. 472 (7), 739–754. 10.1007/s00424-020-02368-3 32222817 PMC7351834

[B8] Hirano M. Kokunai Y. Nagai A. Nakamura Y. Saigoh K. Kusunoki S. (2011). A Novel Mutation in the Calcium Channel Gene in a Family with Hypokalemic Periodic Paralysis. J. Neurol. Sci. 309 (1-2), 9–11. 10.1016/j.jns.2011.07.046 21855088

[B9] Houinato D. Laleye A. Adjien C. Adjagba M. Sternberg D. Hilbert P. (2007). Hypokalaemic Periodic Paralysis Due to the CACNA1S R1239H Mutation in a Large African Family. Neuromuscul. Disord. 17 (5), 419–422. 10.1016/j.nmd.2007.01.020 17418573

[B10] Hunter J. M. Ahearn M. E. Balak C. D. Liang W. S. Kurdoglu A. Corneveaux J. J. (2015). Novel Pathogenic Variants and Genes for Myopathies Identified by Whole Exome Sequencing. Mol. Genet. Genomic Med. 3 (4), 283–301. 10.1002/mgg3.142 26247046 PMC4521965

[B11] Jurkat-Rott K. Mitrovic N. Hang C. Kouzmenkine A. Iaizzo P. Herzog J. (2000). Voltage-sensor Sodium Channel Mutations Cause Hypokalemic Periodic Paralysis Type 2 by Enhanced Inactivation and Reduced Current. Proc. Natl. Acad. Sci. 97 (17), 9549–9554. 10.1073/pnas.97.17.9549 10944223 PMC16902

[B12] Kawamura S. Ikeda Y. Tomita K. Watanabe N. Seki K. (2004). A Family of Hypokalemic Periodic Paralysis with CACNA1S Gene Mutation Showing Incomplete Penetrance in Women. Intern. Med. 43 (3), 218–222. 10.2169/internalmedicine.43.218 15098604

[B13] Ke Q. He F. Lu L. Yu P. Jiang Y. Weng C. (2015). The R900S Mutation in CACNA1S Associated with Hypokalemic Periodic Paralysis. Neuromuscul. Disord. 25 (12), 955–958. 10.1016/j.nmd.2015.09.006 26433613

[B14] Ke Q. Luo B. Qi M. Du Y. Wu W. (2013). Gender Differences in Penetrance and Phenotype in Hypokalemic Periodic Paralysis. Muscle Nerve 47 (1), 41–45. 10.1002/mus.23460 23019082

[B15] Ke T. Gomez C. R. Mateus H. E. Castano J. A. Wang Q. K. (2009). Novel CACNA1S Mutation Causes Autosomal Dominant Hypokalemic Periodic Paralysis in a South American Family. J. Hum. Genet. 54 (11), 660–664. 10.1038/jhg.2009.92 19779499

[B16] Kil T.-H. Kim J.-B. (2010). Severe Respiratory Phenotype Caused by a De Novo Arg528Gly Mutation in the CACNA1S Gene in a Patient with Hypokalemic Periodic Paralysis. Eur. J. Paediatric Neurol. 14 (3), 278–281. 10.1016/j.ejpn.2009.08.004 19822448

[B17] Kim M.-K. Lee S.-H. Park M.-S. Kim B.-C. Cho K.-H. Lee M.-C. (2004). Mutation Screening in Korean Hypokalemic Periodic Paralysis Patients: a Novel SCN4A Arg672Cys Mutation. Neuromuscul. Disord. 14 (11), 727–731. 10.1016/j.nmd.2004.07.005 15482957

[B18] Krych M. Biernacka E. K. Ponińska J. Kukla P. Filipecki A. Gajda R. (2017). Andersen-Tawil Syndrome: Clinical Presentation and Predictors of Symptomatic Arrhythmias - Possible Role of Polymorphisms K897T in KCNH2 and H558R in SCN5A Gene. J. Cardiol. 70 (5), 504–510. 10.1016/j.jjcc.2017.01.009 28336205 PMC5607087

[B19] Kubota T. Wu F. Vicart S. Nakaza M. Sternberg D. Watanabe D. (2020). Hypokalaemic Periodic Paralysis with a Charge-Retaining Substitution in the Voltage Sensor. Brain Commun. 2 (2). fcaa103. 10.1093/braincomms/fcaa103 33005891 PMC7519726

[B20] Kurokawa M. Torio M. Ohkubo K. Tocan V. Ohyama N. Toda N. (2020). The Expanding Phenotype of Hypokalemic Periodic Paralysis in a Japanese Family with p.Val876Glu Mutation in CACNA1S. Mol. Genet. Genomic Med. 8 (4), e1175. 10.1002/mgg3.1175 32104981 PMC7196457

[B30] Li F. F. Li Q. Q. Tan Z. X. Zhang S. Y. Liu J. Zhao E. Y. (2012). A Novel Mutation in CACNA1S Gene Associated With Hypokalemic Periodic Paralysis Which has a Gender Difference in the Penetrance. J. Mol. Neurosci. 46 (2), 378–383. 10.1007/s12031-011-9596-1 21845430

[B21] Neame M. T. Wright D. Chandrasekaran S. (2017). Persisting Fatigue and Myalgia as the Presenting Features in a Case of Hypokalaemic Periodic Paralysis. BMJ Case Rep. 2017, bcr. 10.1136/bcr-2017-219991 PMC5614135 28798241

[B22] Proenza C. Wilkens C. Lorenzon N. M. Beam K. G. (2000). A Carboxyl-Terminal Region Important for the Expression and Targeting of the Skeletal Muscle Dihydropyridine Receptor. J. Biol. Chem. 275 (30), 23169–23174. 10.1074/jbc.M003389200 10801875

[B23] Richards S. Aziz N. Aziz N. Bale S. Bick D. Das S. (2015). Standards and Guidelines for the Interpretation of Sequence Variants: a Joint Consensus Recommendation of the American College of Medical Genetics and Genomics and the Association for Molecular Pathology. Genet. Med. 17 (5), 405–423. 10.1038/gim.2015.30 25741868 PMC4544753

[B24] Schartner V. Romero N. B. Donkervoort S. Treves S. Munot P. Pierson T. M. (2017). Dihydropyridine Receptor (DHPR, CACNA1S) Congenital Myopathy. Acta Neuropathol. 133 (4), 517–533. 10.1007/s00401-016-1656-8 28012042

[B25] Shull G. E. Okunade G. Liu L. H. Kozel P. Periasamy M. Lorenz J. N. (2003). Physiological Functions of Plasma Membrane and Intracellular Ca2+Pumps Revealed by Analysis of Null Mutants. Ann. N. Y Acad. Sci. 986, 453–460. 10.1111/j.1749-6632.2003.tb07229.x 12763865

[B26] Statland J. M. Fontaine B. Hanna M. G. Johnson N. E. Kissel J. T. Sansone V. A. (2018). Review of the Diagnosis and Treatment of Periodic Paralysis. Muscle Nerve 57 (4), 522–530. 10.1002/mus.26009 29125635 PMC5867231

[B27] Tan S. V. Suetterlin K. Männikkö R. Matthews E. Hanna M. G. Bostock H. (2020). *In Vivo* assessment of Interictal Sarcolemmal Membrane Properties in Hypokalaemic and Hyperkalaemic Periodic Paralysis. Clin. Neurophysiol. 131 (4), 816–827. 10.1016/j.clinph.2019.12.414 32066100

[B28] Wu F. Mi W. Hernández-Ochoa E. O. Burns D. K. Fu Y. Gray H. F. (2012). A Calcium Channel Mutant Mouse Model of Hypokalemic Periodic Paralysis. J. Clin. Invest. 122 (12), 4580–4591. 10.1172/JCI66091 23187123 PMC3533564

[B29] Yang H. Zhang H. Xing X. (2015). V876E Mutation in CACNA1S Gene Associated with Severe Hypokalemic Periodic Paralysis in a Chinese Woman. J. Formos. Med. Assoc. 114 (4), 377–378. 10.1016/j.jfma.2013.07.007 23948435

