# Supplementary material for: Case Report: A Novel CACNA1S Mutation Associated With Hypokalemic Periodic Paralysis in a Chinese Family
Source: Front Genet. 2021 Oct 29;12:743184. doi: 10.3389/fgene.2021.743184 (PMC8586648; doi:10.3389/fgene.2021.743184)
Supplement: Supplementary file 1 [file Table1.docx]

**Supplementary Table 1.** The endocrine system diseases-related genes list.

| *ABCC8* | *HNF4A* | *KMT2D* | *SLC16A2* | *GHRHR* | *MAP2K1* | *BBS9* | *MPDU1* | *ALG13* | *MAN1B1* |
| --- | --- | --- | --- | --- | --- | --- | --- | --- | --- |
| *GCK* | *HNF1A* | *CYP21A2* | *SLC5A5* | *BTK* | *MAP2K2* | *BBS10* | *ALG12* | *PGM1* | *CDKN1B* |
| *GLIS3* | *NEUROD1* | *CYP11B1* | *TPO* | *STAT5B* | *KRAS* | *TRIM32* | *ALG8* | *MGAT2* | *MEN1* |
| *HNF1B* | *KLF11* | *CYP17A1* | *TG* | *GHSR* | *SOS1* | *BBS12* | *ALG2* | *MOGS(GCS1)* | *RET* |
| *KCNJ11* | *CEL* | *STAR* | *NKX2-5* | *POU1F1* | *RAF1* | *MKS1* | *DPAGT1* | *SLC35C1* | *MAPK8IP1* |
| *PLAGL1* | *PAX4* | *HSD3B2* | *FOXE1* | *LHX4* | *NRAS* | *CEP290* | *ALG1/HMT-1* | *B4GALT1* | *MKRN3* |
| *PTF1A* | *BLK* | *POR* | *THRA* | *LHX3* | *PREPL* | *WDPCP* | *ALG9* | *SLC35A1* | *DUOXA2* |
| *RFX6* | *GLUD1* | *CYP19A1* | *THRB* | *IGF2* | *SOX3* | *SDCCAG8* | *DOLK(DK1)* | *COG7* | *GHR* |
| *SLC19A2* | *HADH* | *NKX2-1* | *CDC73* | *IGF1* | *BBS1* | *LZTFL1* | *RFT1* | *COG1* | *PTPN11* |
| *ZFP57* | *UCP2* | *PAX8* | *CASR* | *IGF1R* | *BBS2* | *TMEM67* | *DPM3* | *COG8* | *BBS7* |
| *INS* | *LHCGR* | *TSHB* | *MEN1* | *PROP1* | *ARL6* | *CCDC28B* | *ALG11* | *COG5* | *ALG3* |
| *PDX1* | *GNAS* | *TSHR* | *GCM2* | *HESX1* | *BBS4* | *PMM2* | *SRD5A3* | *COG4* | *TUSC3* |
| *EIF2AK3* | *KISS1R* | *DUOX2* | *PTH* | *OTX2* | *BBS5* | *MP1* | *DDOST* | *TMEM165* | *DHDDS* |
| *SLC2A2* | *KISS1* | *IYD* | *GH1* | *SOX2* | *MKKS* | *ALG6* | *MAGT1* | *COG6* | *SCN4A* |
| *KCNJ2* | *KCNJ5* | *ATP2A1* |  |  |  |  |  |  |  |
