# Supplementary material for: Case Report: A Novel CACNA1S Mutation Associated With Hypokalemic Periodic Paralysis in a Chinese Family
Source: Front Genet. 2021 Oct 29;12:743184. doi: 10.3389/fgene.2021.743184 (PMC8586648; doi:10.3389/fgene.2021.743184)
Supplement: Supplementary file 2 [file Table2.docx]

| **Supplement table 2**. Supportive diagnostic criteria for HypoKPP |
| --- |
| 1. Recurrent attacks of muscle weakness with documented serum K <3.5 mEq/L |
| 2. One attack of muscle weakness in the proband, and with a relative has episodic weakness with low serum K |
| 3. Three of 6 clinical or laboratory features:  a. Onset in the first or second decade  b. Attack duration (muscle weakness involving 1 or more limbs, predominantly in lower limbs, and the proximal is more serious than the distal) lasted for hours to days  c. Positive triggers (high carbohydrate rich meal, rest after exercise, exposure to hot or cold, stress)  d. Positive family history or a genetic mutation confirmed  e. Positive long exercise test  f. Improvement with potassium supplementation |
| 4. Other causes of hypokalemia (renal, adrenal, thyroid dysfunction; renal tubular acidosis; diuretic and laxative abuse) are excluded |
| 5. Absence of myotonia, except eye lids. |
